# Rapid destruction of the rhodamine B using TiO_2_ photocatalyst in the liquid phase plasma

**DOI:** 10.1186/1752-153X-7-156

**Published:** 2013-09-16

**Authors:** Heon Lee, Sung Hoon Park, Young-Kwon Park, Byung Hoon Kim, Sun-Jae Kim, Sang-Chul Jung

**Affiliations:** 1 Department of Environmental Engineering, Sunchon National University, Sunchon, Jeonnam 540-742, Korea; 2 School of Environmental Engineering, University of Seoul, Seoul 130-743, Korea; 3 Department of Dental Materials, School of Dentistry, Chosun University, Gwangju 501-759, Korea; 4 Faculty of Nanotechnology and Advanced Materials Engineering, Sejong University, Seoul 143-747, Korea

**Keywords:** Liquid phase plasma, Bubbling, TiO_2_, Pulsed discharge, Dyes

## Abstract

**Background:**

Rhodamine B (RhB) is widely used as a colorant in textiles and food stuffs, and is also a well-known water tracer fluorescent. It is harmful to human beings and animals, and causes irritation of the skin, eyes and respiratory tract. The carcinogenicity, reproductive and developmental toxicity, neurotoxicity and chronic toxicity toward humans and animals have been experimentally proven. RhB cannot be effectively removed by biological treatment due to the slow kinetics. Therefore, RhB is chosen as a model pollutant for liquid phase plasma (LPP) treatment in the present investigation.

**Results:**

This paper presents experimental results for the bleaching of RhB from aqueous solutions in the presence of TiO2 photocatalyst with LPP system. Properties of generated plasma were investigated by optical emission spectroscopy methods. The results of electrical-discharge degradation of RhB showed that the decomposition rate increased with the applied voltage, pulse width, and frequency. The oxygen gas addition to reactant solution increases the degradation rate by active oxygen species. The RhB decomposition rate was shown to increase with the TiO2 particle dosage.

**Conclusion:**

This work presents the conclusions on the photocatalytic oxidation of RhB, as a function of plasma conditions, oxygen gas bubbling as well as TiO2 particle dosage. We knew that using the liquid phase plasma system with TiO2 photocatalyst at high speed we could remove the organic matter in the water.

## Introduction

Due to dyeing of fabric, wastewater from many textile industries contains color and as such cannot be disposed into the environment. Traditional methods for dye removal include biological treatment [1], coagulation [2], filtration [3] and adsorption [4]; however, because of high dye concentrations and the increased stability of synthetic dyes, these methods are becoming less effective for the treatment of colored industry effluents [5,6]. To overcome the problems associated with these traditional methods of dye removal, attention has been focused on advanced oxidation processes (AOPs). AOPs have been developed to degrade biorefractory organics in drinking water and industry effluents [7,8]. AOPs employ a high oxidation-potential source to produce the primary oxidant species, hydroxyl radicals, which react rapidly and unselectively with most organic compounds [9]. Recently, glow discharge in liquid phase has been used to degrade organic pollutants in water [10,11], because not only hydroxyl radicals but also atomic oxygen having a high oxidation potential can be produced in this way. Another application of TiO_2_ photocatalyst in AOPs water treatment has been investigated widely [12,13]. There are still many problems yet to be solved, however, in application of TiO_2_ photocatalyst in the treatment of non-biodegradable materials. First, photocatalysis has usually been used in air pollutants treatment because it is suitable for treatment of low-concentration pollutants. Concentrations of water pollutants, however, are much higher than those of air pollutants. Thus, their treatment by photocatalysis is difficult compared to that of air pollutants. Second, polluted water has high turbidity, hence low transparency, hindering activation of photocatalysts by ultraviolet (UV) rays. Third, the amount of oxygen available for photocatalytic oxidation is very low in water compared to in air. Due to these reasons, photocatalytic oxidation of water pollutants has not received large attention thus far.

RhB is widely used as a colorant in textiles and food stuffs, and is also a well-known water tracer fluorescent [14]. It is harmful to human beings and animals, and causes irritation of the skin, eyes and respiratory tract. The carcinogenicity, reproductive and developmental toxicity, neurotoxicity and chronic toxicity toward humans and animals have been experimentally proven [15,16]. RhB cannot be effectively removed by biological treatment due to the slow kinetics. Therefore, RhB is chosen as a model pollutant for liquid phase plasma (LPP) treatment in the present investigation. This paper presents experimental results for the bleaching of RhB from aqueous solutions in the presence of plasma generated by a bipolar pulsed discharge system added TiO_2_ photocatalyst. Properties of generated plasma were investigated by electrical and optical emission spectroscopy methods. The effects of plasma conditions, addition of oxygen bubbles, and TiO_2_ nanoparticle dosage were investigated.

## Materials and methods

In this study, the decomposition activity was investigated with the RhB (C_28_H_31_ClN_2_O_3_) in its aqueous solution. High purity grade RhB was purchased from Daejung Chemical & metals Co.. RhB aqueous solution 300 ml with the concentration of 1.3 × 10^-2^ mM was prepared. RhB concentration was determined from the absorbance measured by a spectrophotometer (UV-1601, Shimadzu) at 550-nm wavelength. The photocatalyst was Degussa P-25 TiO_2_ (powder, specific surface area 53 m^2^ g^−1^ by the Brunauer-Emmett-Teller method, particle size 20–30 nm by Transmission electron microscopy, composition 83% anatase and 17% rutile by X-ray diffraction).

**Figure 1:**
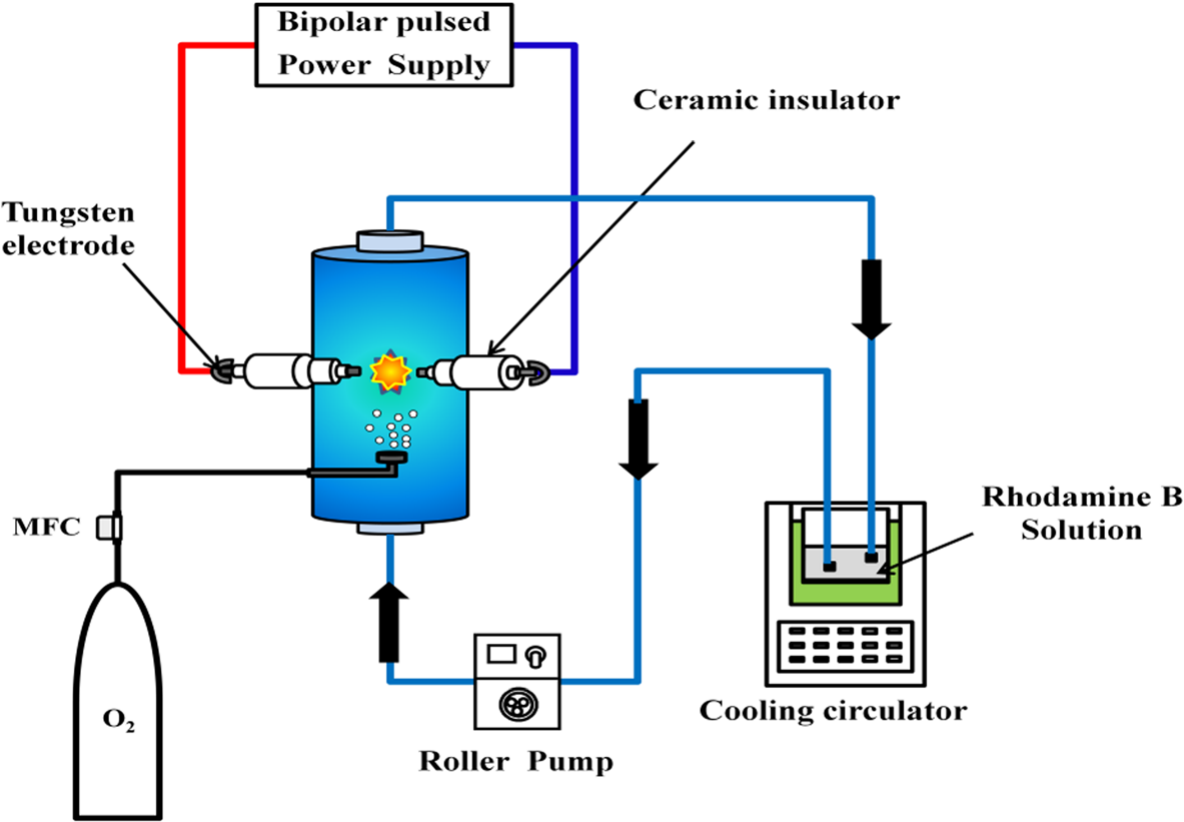
Schematic of the liquid phase plasma treatment system with annular tube type reactor.

A schematic diagram of the experimental device used in this work is shown in Figure 1. The pulsed electric discharge was generated by a needle-to-needle electrode system in a annular tube type reactor (outer diameter of 40 mm and height of 80 mm). Tungsten electrodes (ϕ2 mm, 99.95% purity, T.T.M Korea Co.) with a ceramic insulator coating were used with an interelectrode gap of 0.3 mm. In the case of the system with oxygen bubbling, a glass gas nozzle placed 10 mm below the discharge zone was used. Mass-flow controllers modulated the oxygen gas flow rates set at 100–500 cc/min. The reactant solutions were thermally equilibrated in a cold water bath at 298 K and then circulated into the reactor using a roller pump. In this study, a high-frequency bipolar pulse power supply (Nano technology lnc., NTI-500 W) was utilized to generate pulsed electrical plasma discharge directly in liquid phase. The reason to employ a bipolar pulse system comes from the expectation of symmetrical plasma generation and immediate cleaning of the tip of both electrodes from any possible products adjacent to either cathode or anode, and hence maintenance of stable operation conditions. The ranges of applied voltage, pulse width, and frequency were 230 ~ 250 V, 3 ~ 5 μs, and 25 ~ 30 kHz, respectively.

## Results and discussion

### Optical emission spectroscopy

**Figure 2:**
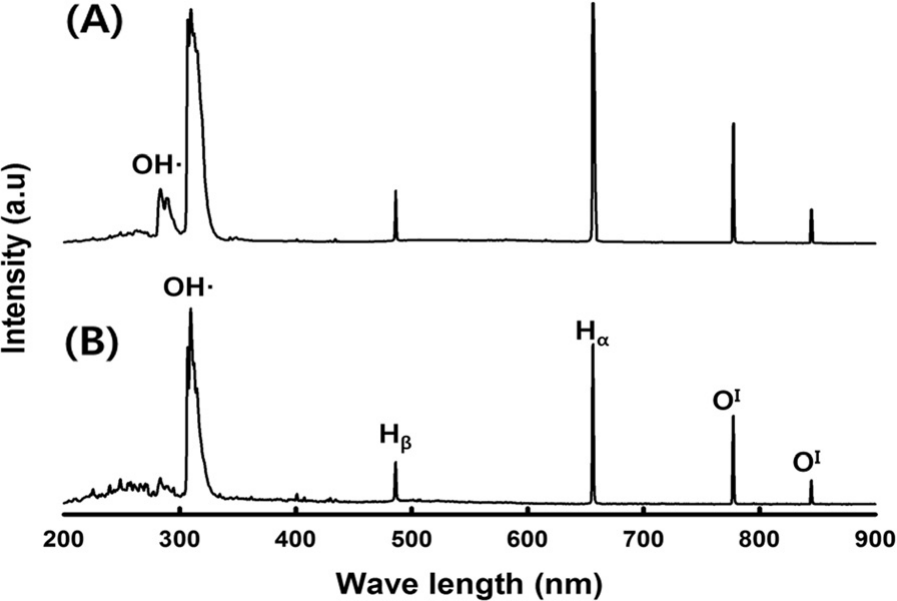
Spatially and temporally integrated emission spectra for the pulsed electric discharge; (A) with oxygen bubbling and (B) without oxygen bubbling.

Similar to gas, liquid has irregular arrangement of molecules. On the other hand, liquid has as many molecules in a unit volume as solid although the molecules are not fixed. It is expected that many kinds of chemically active species would be generated in LPP by the electrical discharge system. Therefore, it is necessary to identify the chemical species produced by the discharge and to investigate the effects of the active species. The spectroscopic measurements were performed during the electrical discharges for the tungsten electrodes using an AvaSpec-3648 Fiber optical spectrometer (Avantes). The emission was detected with an optical fiber coming out perpendicularly to the axis of the electrodes. The experimental conditions were kept constant during the spectra acquisition (discharge voltage 250 V, frequency 30 kHz, and pulse width 5 μs). The optical emission spectra acquired during the discharge with (A) and without (B) oxygen bubbling are shown in Figure 2. The excited states of atomic hydrogen (H_α_ at 656.3 nm and H_β_ at 486.1 nm) and atomic oxygen (3p^5^P → 3s^5^S^0^ at 777 nm and 3p^3^P → 3s^3^S^0^ at 844 nm) as well as the molecular bands of the hydroxyl radical OH (at 283 and 309 nm) were detected in the emission spectra. LPP provides extremely rapid reactions caused by activated chemical species and radicals under high pressure [17]. The hydrogen radicals produced in LPP can be applied to synthesis of metal particles by the liquid-phase chemical reduction methods [18], whereas the atomic oxygen and hydroxyl radicals can be used for oxidation of organic compounds as in this study. Detection of the hydroxyl radical at 283 nm was acquired only when oxygen gas was bubbled (A). Therefore, it is expected that oxygen gas bubbling would enhance the decomposition of organic compounds in LPP.

### Effect of impressed voltage

**Figure 3:**
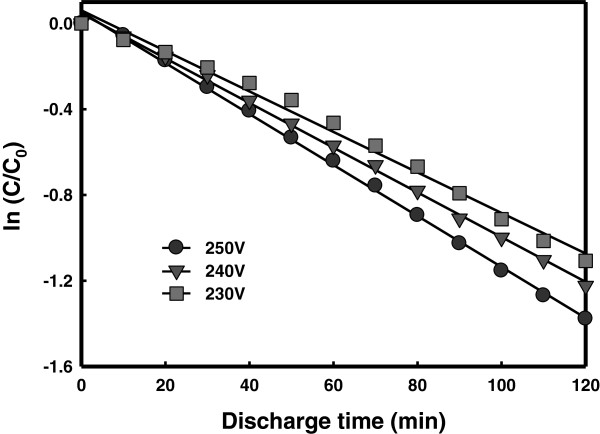
Decomposition of RhB with different impressed voltage levels.

The effects of impressed voltage on the generation of LPP and the decomposition of RhB were investigated. The voltage level was increased stepwise by 10 V from a sufficiently low level using a high-frequency bipolar pulse power supply. The LPP was generated due to breakdown occurring at the impressed voltage of 230 V or higher when the pulse width was 5 μs and the frequency was 30 kHz. At or below 220 V only bubbles were produced without the occurrence of breakdown. At a higher impressed voltage level (≥ 230 V), plasma was generated together with breakdown, while the glare of plasma was more intense with a higher impressed voltage level. Figure 3 compares the results of the decomposition experiments of RhB using LPP treatment performed with different impressed voltage levels. The decomposition rate of RhB was shown to increase with the impressed voltage level. This result indicates that a higher impressed voltage causes the more intense plasma producing the more hydroxyl radicals and leads to a higher decomposition efficiency of RhB.

### Effect of pulse width

**Figure 4:**
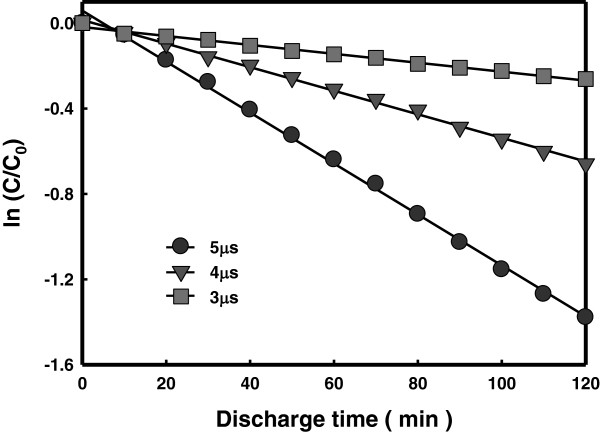
Decomposition of RhB with different pulse width.

Generally, when direct current is used in liquid phase, melting of the electrodes happens due to collisions of ions on the electrodes and heat transfer from surrounding hot gas, causing electrode loss and water pollution problems. In this study, a high-frequency bipolar pulse power supply was used to control the pulse width of the impressed voltage at a μs level. With this short pulse voltage, acceleration of heavy ions, resulting heating of electrodes and solution, and the electrode loss were effectively inhibited. The effect of the pulse width level on the decomposition rate of RhB in the liquid phase plasma process was examined. Figure 4 compares the concentration decays obtained with different pulse width levels used in the liquid phase plasma treatment. The impressed voltage and the frequency were 250 V and 30 kHz, respectively. The decomposition rate of RhB was shown to be higher at a larger pulse width. A larger pulse width of the impressed voltage generally leads to a larger plasma generation time per unit time. Therefore, it is believed that a larger pulse width led to production of hydroxyl radicals for a longer time, resulting in a higher decomposition efficiency of RhB in this study.

### Effect of frequency

**Figure 5:**
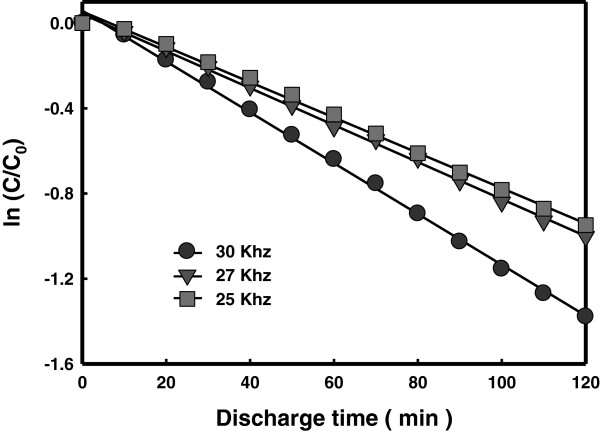
Decomposition of RhB obtained with different frequencies.

The preliminary experiments for plasma generation showed that electric discharge did not occur when the pulse width and the frequency of the impressed voltage were small because the joule heat was not enough to provoke vaporization of the solution. As the pulse width and the frequency of the impressed voltage were increased, however, the size of the bubbles produced between the electrodes by joule heating increased leading to outbreak of the electric discharge. The effect of the frequency on the decomposition rate of RhB in the LPP process is shown in Figure 5. The impressed voltage and the pulse width were 250 V and 5 μs, respectively. A higher frequency led to a higher decomposition rate of RhB, which is attributed to a production of a larger amount of hydroxyl radicals at a higher frequency.

### Effect of oxygen gas bubbling

**Figure 6:**
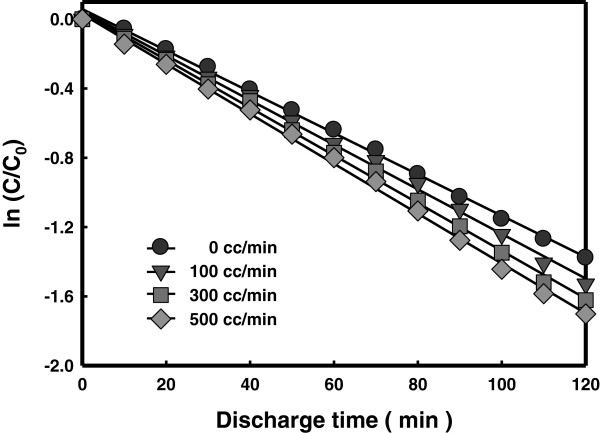
Decomposition of RhB with different oxygen gas bubbling rate.

Water treatment is an advanced technology that oxidizes organic and toxic compounds by producing strong oxidants, OH radicals, as intermediate products. As various new kinds of water pollution sources appear, however, non-biodegradable materials that would not be degraded easily even have recently been reported. In particular, water treatment using TiO_2_ photocatalysts has a disadvantage of using small amount of oxygen dissolved in water. In this study, the effect of provision of additional oxygen gas was investigated. Figure 6 compares the RhB decomposition results obtained with different levels of oxygen gas bubbling. The RhB decomposition rate increased with the oxygen gas bubbling rate. Producing electrical discharges in water in the presence of externally introduced gas bubbles improves the energy balance with more energy used to produce chemically active species [19]. It can be deduced from this result that oxygen added in LPP treatments can help increase the degradation rate by playing a role as auxiliary oxidants.

Table 1 shows the OES counts for hydroxyl radicals obtained with different O_2_ gas flow rates, measured by the optical spectrometer. The OES count increased with increasing oxygen gas flow rate. In particular, the OES count at 283 nm obtained with 500 cc/min oxygen flow rate was about three times that obtained with no oxygen gas. Hydroxyl radicals are known to have strong oxidizing power, which is believed to have contributed to the enhanced decomposition of RhB, as was shown in Figure 6.

**Table 1:** **Detected hydroxyl radicals in the emission spectra obtained at different O**_**2 **_**gas flow rate**

**O**_**2 **_**gas flow rate [ cc/min ]**	**OES counts [ a.u. ]**	
	**283 nm**	**309 nm**
0	928	9768
100	1238	10038
300	2140	10924
500	2737	11716

### Effect of TiO_2_ particle dosages

**Figure 7:**
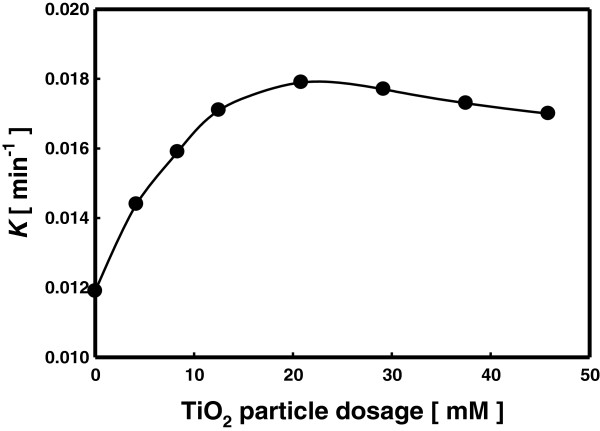
**Effect of TiO**_**2 **_**particle dosage on the decomposition of RhB with LPP method.**

Figure 7 compares the decomposition reaction rate constants obtained with different amounts of TiO_2_ particle dosage. The RhB decomposition rate was shown to increase with the TiO_2_ particle dosage until the dosage exceed a threshold value (about 20 mM). The TiO_2_ photocatalyst is activated by ultraviolet (UV) to produce oxidizing agents such as OH radicals. The result shown in Figure 7 indicates that in this study the TiO_2_ particles were activated by UV emitted from the plasma to promote the decomposition reaction of RhB.

## Conclusions

The following conclusions were inferred from the results of decomposition of RhB via TiO_2_ photocatalyst and LPP system:

(1) The excited states of atomic hydrogen and atomic oxygen as well as the molecular bands of the hydroxyl radical were detected in the emission spectra and the hydroxyl radical at 283 nm was acquired only when oxygen gas was bubbled.

(2) The results of LPP degradation of RhB showed that the decomposition rate increased with the applied voltage, pulse width, and frequency.

(3) The oxygen gas addition to reactant solution increases the degradation rate by active oxygen species, which is generated from adding oxygen gas.

(4) The RhB decomposition rate was shown to increase with the TiO_2_ particle dosage.

## Competing interests

The authors declare that they have no competing interests.

## Authors’ contributions

LH and PSH carried out the main part of experiment and drafted the manuscript. PYK measured and analyzed the optical emission spectra. KBH measured the UV absorbance and calculated the degradation rate constant. KSJ provided assistance with the data analysis and investigated the relationship between variables and results. JSC coordinated the experimental design and contributed the manuscript writing. All the authors read and approved the final manuscript.
